# Comparison of Drought Stress Response and Gene Expression between a GM Maize Variety and a Near-Isogenic Non-GM Variety

**DOI:** 10.1371/journal.pone.0117073

**Published:** 2015-02-18

**Authors:** Mariolina Gullì, Elisabetta Salvatori, Lina Fusaro, Claudia Pellacani, Fausto Manes, Nelson Marmiroli

**Affiliations:** 1 Department of Life Sciences, University of Parma, Parco Area delle Scienze 11a, 43124 Parma, Italy; 2 Department of Environmental Biology, Sapienza University of Rome, P.le Aldo Moro 5, 00185 Rome, Italy; Kansas State University, UNITED STATES

## Abstract

Maize MON810, grown and commercialised worldwide, is the only cultivated GM event in the EU. Maize MON810, variety DKC6575, and the corresponding near-isogenic line Tietar were studied in different growth conditions, to compare their behaviour in response to drought. Main photosynthetic parameters were significantly affected by drought stress in both GM and non-GM varieties to a similar extent. Though DKC6575 (GM) had a greater sensitivity in the early phase of stress response as compared with Tietar (non-GM), after six days of stress they behaved similarly, and both varieties recovered from stress damage. Profiling gene expression in water deficit regimes and in a generalised drought stress condition showed an up-regulation of many stress-responsive genes, but a greater number of differentially expressed genes was observed in Tietar, with genes belonging to transcription factor families and genes encoding heat shock proteins, late embryogenesis abundant proteins and detoxification enzymes. Since induction of these genes have been indicated from the literature as typical of stress responses, their activation in Tietar rather than in DKC6575 may be reminiscent of a more efficient response to drought. DKC6575 was also analysed for the expression of the transgene *CryIAb* (encoding the delta-endotoxin insecticidal protein) in water deficit conditions. In all the experiments, the *CryIAb* transcript was not influenced by drought stress, but was expressed at a constant level. This suggests that though possessing a different pattern of sensitivity to stress, the GM variety maintains the same expression level for the transgene.

## Introduction

Environmental sustainability and competitiveness in the production of commodities and in the economical evaluation of inputs and outputs, are among the main challenges of modern agriculture. A case is the extreme exploitation of water resources, which involves a trade-off between economic development and sustainability. Particular attention for water saving in agriculture is certainly needed because of: i) its increasing demand for human consumption; ii) its increasing use for industry; iii) the need to protect water stocks and to maintain the aquatic environments.

Maize culture is strategic both for human and animal nutrition, as well as for the industrial production of starch, oil, and bio-fuels. Over the past decade its demand as livestock feed has grown tremendously due to the rapid economic growth in Asia, the Middle East and Latin America [1], but increased also its demand as an industrial raw material due to its use in bio-ethanol programmes. For these reasons maize is referred to as a commodity for FFP (Food, Feed and Processing).

The maize growth cycle is restricted to spring and summer, and characterized by a high water demand. It has been calculated that 1 ha of maize uses up to 10 million m^3^ of water. Although varieties with a better environmental adaptability have been produced, and there are examples of maize cropped under deficit irrigation or even in rainfed conditions, the mainstream in maize cultivation still follows the rule of intense watering. Therefore, any reduction of water availability, together with exacerbation of other abiotic and biotic stresses that may occur as a consequence of climate changes, could affect negatively maize growth and its productivity [2]. This risk is particularly high in Southern Europe and in all the Mediterranean regions, where climate variability and extreme events are expected to increase [3].

The development of an improved maize germplasm through engineered modifications (GM maize) have the potential to offset some of the predicted climate change-related crop yield losses. In particular, a higher stress tolerance and a lower water demand are common claims both for commercial hybrids and for their GM varieties that have been positively evaluated within the European Commission (Commission Regulation EC 258/97; EC 50/2000; EC 1829/2003; EC 1830/2003, reviewed in [4]), and released for cultivation within the EU: Bt-176, MON810, Bt-11, GA21.

**Fig 1:**
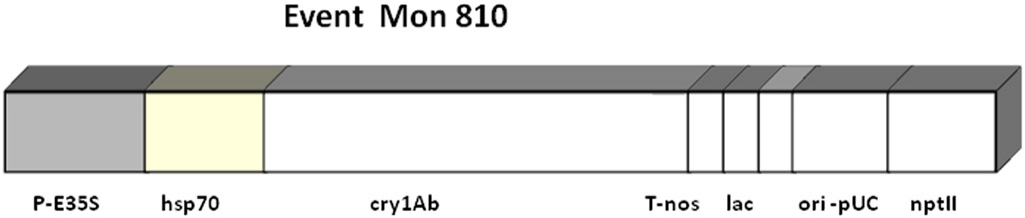
Schematic representation of the *Cry1Ab* transgene cassette, used in the making of MON810.

Among these, MON 810 (Fig. 1), which bears resistance to European corn borer, is the most cultivated GM event in the EU. It contains a stable, genome-integrated plant expression cassette comprised of the cauliflower mosaic virus 35S promoter and HSP70 maize intron sequence, driving the expression of a synthetic *CryIAb* gene. The 3′ truncated *CryIAb* gene codes for a delta-endotoxin that acts as a potent and highly specific insecticide [5]. Expression of the genes introduced by genetic modification has been sufficiently analysed and the stability of the genetic transformation has been demonstrated over several generations [6].

Within the EU the notification procedure for realising transgenic plants for cultivation and for FFP requires information regarding the molecular characteristics of the transgene, and its behaviour in several trials and in different environments. However, little or no information is available on the stress level present in the testing environments though it would be very interesting to know the behaviour of the transgene in defined and measurable stress condition. It is known that GM plants can differ from their isogenic plants in several agronomic and ecophysiological traits from their isogenic plants, since the introduction of transgenes may cause unintended effects [7]. In fact environmental stress could, in their turn, modify gene expression in general, including the transgene expression [8]. In the case of maize, water stress would certainly be the most relevant environmental parameter.

Interplay has recently been demonstrated of water and insect stress with plant injury, including fungal inoculation, and yield, and their interactions with Bt transgenes. The relevance of knowing more of these interactions when considering strategies for Bt cultivation under water stress is paramount [9].

The aim of the present study was to assess the effect of water deficit on physiological parameters, on the global transcriptional pattern, and on *CryIAb* gene expression in the GM maize variety DKC6575, compared with its near-isogenic non-GM Tietar. In particular, the following hypotheses were tested: i) the GM and the non-GM variety do not differ in their drought stress response, both at the ecophysiological and molecular levels; ii) the expression of the Bt transgene in water limiting conditions is not affected by water availability. The abovementioned hypotheses were analysed both in the field and in controlled growth chamber conditions.

## Materials and Methods

### Ethics statement

No animal trial was conducted. All experiments were performed in laboratories at the University of Parma and at Sapienza University of Rome. The experimentation was planned within the national project PRIN 2006 and was performed in a private and confined field. The competent authorities were informed about the measures of containment and of disposal adopted. Plants were grown at the University of Parma and in a closed “walk-in” chamber at Sapienza University of Rome, and all plant material residues and soil were destroyed after the study was finished.

### Plant material and plant growth conditions

Maize (*Zea mays* L.) seeds of commercial variety of the YieldGard MON810 event DK6575 (Monsanto), and the near-isogenic Tietar, were obtained from Spanish purchasers. Seeds of DKC6575 were tested for the presence of the transgene using an event-specific real-time PCR, and using as a positive comparator certified reference material from IRMM (Institute of Reference Materials and Measurements, Geel, Belgium). Genomic DNA from seeds of Tietar was analysed with the same event-specific test, giving no amplification by real-time PCR (data not shown).

Field experiment

Seeds of Tietar and DKC6575 were grown in an authorized and totally segregated field in Parma (Italy) 44.80°N 10.32°E, with a typical continental climate; the soil type in this area is coarse-loamy, mixed, calcareous. The field under study was divided into three 40 m^2^ plots, 3 rows wide (row spacing 0.60 m) and 24 m long. Seeds were sown at a density of 80,000 plants/ha and were treated following standard agricultural practices. Each plot was fully irrigated until plants reached the vegetative six-leaf (V6) stage; from V6 to harvest they were irrigated differently to apply a drought stress: plot 1 was irrigated at 100% field capacity (control); plot 2 was irrigated at 25% field capacity (stress 1); plot 3 was not irrigated (0% field capacity, stress 2). Stress level was evaluated according to the suggestions in the web site http://maizedoctor.cimmyt.org.

Growth chamber experiment

Seeds of Tietar and of DKC6575 were grown in a closed “walk-in” chamber (2.5 m × 3.9 m × 3.0 m height) [10]. Fourteen plants for each hybrid were grown in 18 L pots, and maintained at 28/25°C (day/night), 50% relative humidity, photoperiod 14h (PAR 700 μE m^-2^s^-1^). All plants were watered daily to field capacity with a sub-irrigation system, until the complete development of the 8th-9th leaf (V8). At this stage, plants were randomly divided into four experimental sets: seven plants of each genotype were watered at 100% during the whole experiment (control set), while another seven plants of each genotype were not irrigated for six days (drought stressed set). Soil water content was monitored gravimetrically, by weighing each pot daily.

### Ecophysiological measurements

In all experiments, net photosynthesis (A, μmol CO_2_ m^-2^ s^-1^), leaf transpiration (E, mmol H_2_O m^-2^ s^-1^), stomatal conductance (g_s_, mmol H_2_O m^-2^ s^-1^) and sub-stomatal CO_2_ concentration (Ci, ppm) were simultaneously measured with a portable infrared gas analyzer (CIRAS 2, PP Systems, Amesbury, MA, USA). The ratio between substomatal and external CO_2_ concentration (Ci/Ca), as well as the water use efficiency (WUE = A/g_s_, μmol CO_2_ mmol H_2_O^-1^) were also calculated.

Chlorophyll “a” (Chl a) fluorescence was measured using the Fluorescence Monitoring System (FMSII, Hansatech, UK) instrument. The maximum quantum yield of PSII was evaluated on dark-adapted leaves as F_v_/F_m_ = (F_m_−F_0_)/F_m_) where F_0_ is the basal fluorescence, F_v_ the variable fluorescence and F_m_ is the maximum fluorescence. The effective quantum efficiency ΦPSII was evaluated as (F_m′_−F_s_)/F_m′_, where F_s_ is the steady state fluorescence and F_m′_ is the maximum fluorescence measured in the light. Photochemical (qP) and non-photochemical (qNP) quenchings were also derived [11].

Sampling scheme for field experiment

**Fig 2:**
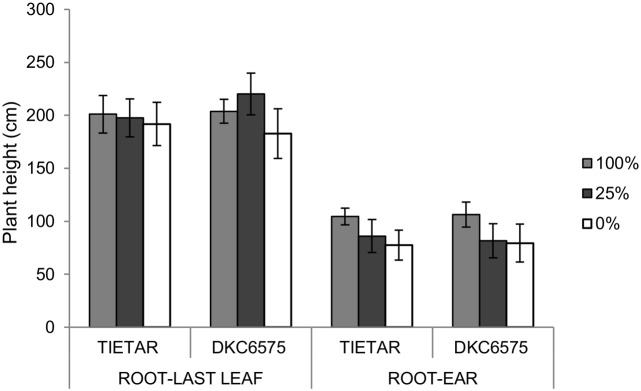
Heights (cm), measured in plants grown in the field in control conditions (100% field capacity) and in stressed conditions (25% and 0% field capacity).

Measurements were made on plot 1 (100% field capacity, control) and plot 3 (0% field capacity, stress 2) at the silking (R1) stage, after 20 d from the beginning of the drought treatment (T2). Measurements were made in the morning from 7:00 to 13:00 GMT+1 on 4 representative individuals per treatment, randomly located in the experimental plots. Three leaves per plants were measured, as described above. At the end of the experiment, plant height was evaluated measuring the distance from the root to the last leaf, and root to ear (Fig. 2).

Sampling scheme for growth chamber experiment

Physiological parameters were measured at full irrigation of all sets (0I, 0II) during the drought treatment (SI, SII, SIII) corresponding to 4, 5 and 6 d from the last irrigation of the drought stressed sets, and after the re-irrigation of the drought stressed sets (RI, RII corresponding to 2 and 4 d after re-irrigation). Two leaves per plants were sampled: the last developing and the first fully developed leaves from the top of the plant. At the 0I and SIII stages, the response of net photosynthesis to the variation of substomatal CO_2_ concentration (A vs Ci response curve) was measured on two plants per genotype and treatment, on the same leaves sampled for steady state gas exchanges. The A/Ci curves were constructed according to Long and Bernacchi [12]. The in vivo apparent Rubisco activity (V_c,max,_ mol m^-2^s^-1^) was derived from the angular coefficient of the linear part of the curve. The CO_2_ compensation point Γ (ppm) was also derived. Data at very low [CO_2_], that could be limited by Rubisco deactivation, were excluded from the analysis [13]. At the end of the experiment, all plants were harvested and dried at 80°C until constant weight was reached. The amount of dry biomass of leaves, stems and roots produced in each treatment was then determined by means of an electronic balance (Europe 1700, Gibertini, Italy).

### Profiling Gene Expression

Database search and primer design

Sequences of *Z*. *mays* encoding the actin binding protein (*Zmabp3*), the 18S small subunit of ribosomal RNA (*Zm18SRNA)*, dehydrin (*Zmdhn1*), and the transgene (*CryIAb*) were selected from GenBank. Specific primers and probes for quantitative Real Time Reverse Transcriptase PCR (qRT-PCR) were designed using ‘‘Primer Express 2” (Applied Biosystems, Foster City, CA) (Table 1). All primers were obtained from MWG (Ebersberg, Germany) and probes from Applied Biosystems.

**Table 1:** Primer and probe sequences utilised for the expression analysis of the target genes by qRT-PCR.

Target gene		Primer sequence*		Probe sequence*
Name	Accession n°	Forward	Reverse	
Zmdhn1	X15290	AGGAAGAAGGGAATCAAGGAGAAGA	CGTGCTGGTCGTCCTTGT	**FAM**-CTCCGGGCAGCTTC-**MGB-NFQ**
Cry1Ab	AM750006.1	TGCCTCGACGAGAAGAAGGA	CTGAGACGCTTGGCATGCT	**VIC**-CTGTCCGAGAAGGTG-**MGB-NFQ**
Zm18S rRNA	AF168884	CCGGCGACGCATCATT	GGCCCCTATATCCTACCATCGA	**FAM**-AAATTTCTGCCCTATCAAC-**MGB-NFQ**
ZmAbp3	X97726.1	TGTGAACGATGAGTGCATGCT	CGGTGCAGCCTCTTCGA	**FAM**-AAGTTTGGCGAGCTGC-**MGB-NFQ**

*Sequences are given in 5’- 3’ order

Evaluation of primer efficiency

Each primer pair utilized in qRT-PCR was tested for efficiency and specificity as previously described [14]. Efficiency (E) was determined with the standard curve methods and this gave: E = 1.01 for *Zm18SRNA* (R^2^ = 0.988), E = 0.95 for *Zmdhn1* (R^2^ = 0.988), E = 1.01 for *CryIAb* (R^2^ = 0.997), E = 1.08 for *Zmabp3* (R^2^ = 0.990).

Sampling scheme for gene expression profiling in field experiment

Samples of leaves were collected from plants of plot 1 and of plot 3 at the vegetative six-leaf stage (V6) (T0 stage); at the vegetative eight-leaf stage (V8) (T1 stage) and at silking (R1) (T2 stage), all at the same hour of the day, immediately frozen in liquid nitrogen and stored at -80°C. Each sample consisted of 10 cm-long leaf portions, obtained by discarding the 5 cm apical portion and removing the central vein from the usable section. Samples were collected from some plants of each plot, carefully checked for the absence of infections and other lesions. Three biological replicates were taken per maize variety and growth stage, each grown in a different micro-plot.

Sampling scheme for gene expression profiling in growth chamber experiment

Gene expression was measured at full irrigation of all sets (0I, 0II) during the water treatment (SI, SII, corresponding to 4, and 5 d from the last irrigation of the drought stressed sets), and after the re-irrigation of the drought stressed sets (RI, RII corresponding to 2 and 4 d after re-irrigation). Two leaves per plants were sampled, corresponding to the last developing leaf and the first fully-developed leaf from the top of the plant.

RNA purification and qRT-PCR analyses

Total RNA was extracted from all samples using the RNeasy kit (Qiagen, Hilden, Germany), RNA concentration, integrity and purity were checked as previously described [15]. Total RNA samples were treated with the QuantiTect Reverse Transcription Kit (Qiagen, Hilden, Germany) to eliminate residual genomic DNA and to synthesize first-strand cDNA according to the manufacturer’s instructions. Twenty ng of first strand cDNA were used in a total reaction volume of 25 μL with 1X TaqMan Universal Mastermix (Applied Biosystems), 250 nM of probe, 200 nM each of gene specific primers. Reactions were performed on an ABI Prism 7900HT (Applied Biosystems) instrument with the following conditions: 50°C for 2 min, 95°C for 10 min followed by 40 cycles of 95°C for 15 s, and 60°C for 1 min. Each sample was amplified in triplicate, and each experiment was repeated twice.

Microarray hybridization and analyses

The Gene Chip Maize Genome Array (Affymetrix, Santa Clara, CA, USA) was utilised for profiling transcriptional differences between a MON810 GM maize and the corresponding near-isogenic variety (DKC6575 vs. Tietar) in the following field experiments, which seemed the most representative for its physiological response:

a) fully irrigated T1 (Cont T1); b) fully irrigated T2 (Cont T2); c) drought stress (0% field capacity) condition (Stress T2).

The Maize Genome Array has 17,555 probe sets to interrogate approximately 14,850 *Z*. *mays* transcripts, which represent 13,339 genes, in comparison with a total estimated of 21,298 (assembly B73 RefGen_v2; ZmaysB73_wgs_1.0), representing 63% genome coverage. Total RNA was extracted using the Spectrum Plant Total RNA Kit (Sigma, St. Louis, MO, USA) according to the manufacturer’s instructions. RNA concentration was quantified by UV absorption at 260 nm using a Nanodrop ND100 spectrophotometer (Nanodrop Technologies, Wilmington, De, USA). The integrity of RNA samples was assessed by capillary electrophoresis using a Bioanalyser 2100 (Agilent Technologies, Palo Alto, CA, USA). From 500ng of each RNA samples, first strand cDNA was synthesized using the GeneChip T7-oligo(dT) Promoter Primer kit followed by the cDNA synthesis using SuperScriptII (Invitrogen, Life Technologies) according to the eukaryotic sample processing protocol. The complementary DNA (cDNA) was used as a template for in vitro transcription using the ENZO BioArray High Yield RNA Transcript Labeling Kit (Affymetrix) to obtain biotin labelled cRNA. After cleanup and spectrophotometric quantification the biotinylated cRNA was fragmented into short sequences (100 nt) and used to hybridize to the GeneChip Maize Genome Array (Affymetrix) in the GeneChip Hybridisation Oven (Affymetrix) for 16 h at 45°C. Chips were subsequently washed and fluorescently labeled with phycoerythrin in the GeneChip Fluidics Station 450 and fluorescence was quantified using the GeneChip 3000 scanner device.

### Statistical and Bioinformatic analysis

Ecophysiological data analysis

Statistical analyses of gas exchanges, chlorophyll fluorescence and biomass data collected both in field and chamber experiments were made using Statistica 7 software package (StatSoft, Inc. Tulsa, OK, USA). A two-way Analysis of Variance (ANOVA, p<0.05), was applied, followed by the Newman–Keuls test, taking into consideration variety and treatment as discriminant factors. Normality and homogeneity of variance (Levene’s test) requirements were previously tested, and data transformed when necessary. Data in figures and tables are presented as percentage variation of the water stress treatment (irrigation 0% of field capacity) with respect to the control (irrigation 100% of field capacity).

qRT-PCR data analysis

The qRT-PCR results were analysed using the 2^-ΔΔCt^ method [16] to evaluate the amount of target contained in each sample. *Zmdhn1* and *Cry1A* transcript level were normalized with respect to *Zmabp3* or to *Zm18SRNA*. Relative quantification of expression of the target genes was evaluated in relation to T0 samples (calibrator). Data analyses were performed with Data Assist v2.0 (Applied Biosystems). The most stable transcript was chosen using the geNorm v 3.4 statistical algorithm [17]. *Zmabp3* was used as reference gene for data normalisation. Expression data were validated by statistical analysis using a two-way Analysis of Variance (ANOVA, p<0.05), followed by the Tukey test. Data in figures are presented as mean ± standard deviation.

Microarray data analysis

The Robust Multichip Average (RMA) software [18] was used to extract the data. The Partek Genomics Suite 6.5 (Partek Genomics) was used for data analyses. Functional annotation of the transcripts differentially expressed in stress conditions for both varieties was obtained using the functional classification in the mapping file that structure the maize genes from the Affymetrix maize array into distinct metabolic and cellular components. Gene Ontology (GO) and functional annotation of the list of differentially expressed genes were performed with DAVID software v6.7 [19], [20]. Two-dimensional (2-D) hierarchical clustering of genes on the basis of their expression patterns across multiple experiments was performed using the software Cluster and TreeView, an integrated pair of programs for analysing and visualizing the results of complex microarray experiments [21].

The data presented in this paper have been deposited in NCBI’s Gene Expression Omnibus [22] and are accessible through GEO Series accession number GSE59533 (http://www.ncbi.nlm.nih.gov/geo/query/acc.cgi?acc=GSE59533).

## Results

### Ecophysiological measurements

Field experiment

In both maize varieties, a significant reduction of net photosynthesis, stomatal conductance and leaf transpiration was evident in drought stressed plants after 20 d without irrigation (T2) (Tables 2 and 3). In particular, drought stressed Tietar plants showed a g_s_ reduction of 63.01% in respect to the control, while in drought stressed DKC6575 the reduction of g_s_ was 40.02% compared with the control. Concurrently, Ci/Ca decreased in both varieties (-57.4% in Tietar, -26.1% in DKC6575). As expected, water use efficiency (WUE) significantly increased in drought stressed plants of both varieties.

**Table 2:** Percentage variation in the drought stress treatment (irrigation 0% of field capacity) with respect to the control (irrigation 0% of field capacity) for selected photosynthesis (A = net photosynthesis; g_s_ = stomatal conductance to water vapour; E = leaf transpiration; Ci/Ca = ratio between stomatal and ambient CO_2_ concentration; WUE = Water Use Efficiency), and fluorescence parameters (ΦPSII = effective quantum efficiency of PSII; qP = photochemical quenching; qNP = non photochemical quenching; ETR = electron transport rate; F_v_/F_m_ = maximum quantum yield of PSII), measured on Tietar and DKC6575 at T2 (after 20 d without irrigation) in the field experiment.

Variety	Sampling point	A (%)	g_s_ (%)	E (%)	Ci/Ca (%)	WUE (%)	ΦPSII (%)	qP (%)	qNP (%)	ETR (%)
Tietar	T2	-49.0	-63.0	-55.9	-57.4	16.9	-16.6	-15.7	18.9	-45.4
DKC6575	T2	-27.9	-40.0	-31.3	-26.0	8.9	-0.1	-0.3	0.5	-26.0

**Table 3:** Two-Way ANOVA on selected photosynthesis (A = net photosynthesis; g_s_ = stomatal conductance to water vapour; E = leaf transpiration; Ci/Ca = ratio between stomatal and ambient CO_2_ concentration; WUE = Water Use Efficiency), and fluorescence parameters (ΦPSII = effective quantum efficiency of PSII; qP = photochemical quenching; qNP = non photochemical quenching; ETR = electron transport rate; F_v_/F_m_ = maximum quantum yield of PSII), measured on Tietar and DKC6575 at T2 (after 20 d without irrigation) in the field experiment.

Factor	Sampling point	A	g_s_	E	Ci/Ca	WUE	ΦPSII	qP	qNP	ETR
Variety	T2	**0.000**	**0.000**	**0.000**	0.487	0.317	**0.005**	**0.002**	**0.002**	**0.374**
Drought stress		**0.000**	**0.000**	**0.000**	**0.003**	**0.037**	0.120	0.112	0.112	**0.000**
Variety x Drought stress		0.187	0.313	0.109	0.211	0.502	0.123	0.128	0.128	0.196

Significant (p < 0.05) factors are bold faced.

Similar to the effects of drought stress on gas exchange, chlorophyll “a” fluorescence showed a significant reduction of the electron transport rate (ETR) in drought stressed plants, particularly in Tietar (45.4% lower than in the control). However no significant treatment effect was evident in the effective quantum efficiency ΦPSII, nor in photochemical (qP) and non-photochemical (qNP) quenching (Tables 2 and 3).

Growth chamber experiment

**Fig 3:**
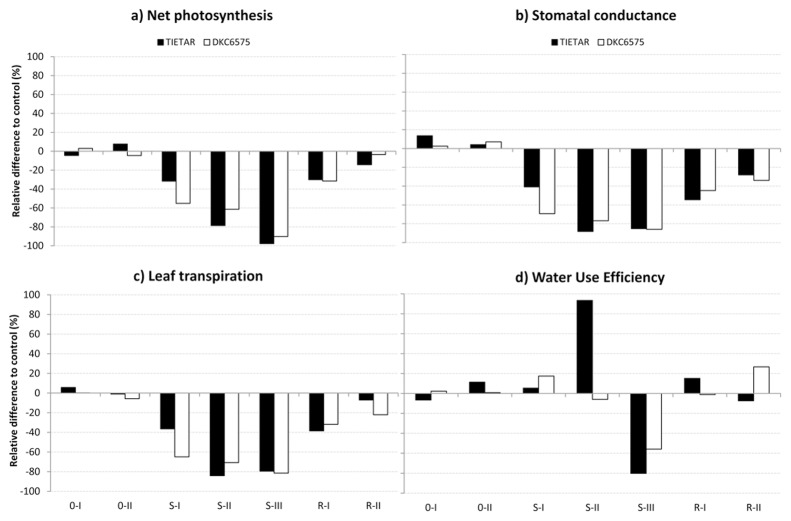
Percentage variation of the drought stress treatment (irrigation 0% of field capacity) in respect to the control (irrigation 100% of field capacity) for gas exchanges parameters measured in the growth chamber experiment. The straight line at the “0” level represents the control, and histograms represent the relative variations of drought stressed plants.

During the period without irrigation, both maize varieties showed a progressive reduction of gas exchange (Fig. 3). However, after 5 d without irrigation (S-II), this reduction was significantly higher in the non-GM Tietar than in the GM DKC6575 (Table 4). Interestingly, at SII Tietar displayed higher WUE, although the difference was not significant due to the increased variability in treated plants (Table 4). At SIII, WUE was significantly reduced in both varieties (80.2% and 55.8% lower than the control for Tietar and DKC6575, respectively) (Fig. 3, Table 4). Net photosynthesis and leaf transpiration recovered completely four days after re-watering (RII), with no difference between the two varieties; only stomatal conductance remained significantly lower in plants that had been stressed, both GM and non-GM variety (33.8% and 28% than the control, respectively) (Fig. 3, Table 3).

**Table 4:** Two-Way ANOVA on gas exchange and chlorophyll fluorescence parameters, for each measurement date in the growth chamber experiment. 0I, 0II = before the beginning of the drought treatment; SI, SII, SIII = corresponding to 4, 5 and 6 d from the last irrigation of the drought stressed sets, respectively; RI, RII = corresponding to 2 and 4 d after re-irrigation of the drought stressed sets.

Factor	SamplingPoint	A	g_s_	E	WUE	ΦPSII	qNP	qP	ETR
Variety	0I	0.381	0.589	0.378	0.526	0.278	0.268	0.268	0.163
Drought stress		0.920	0.490	0.753	0.815	0.347	0.499	0.499	0.944
Variety x Drought stress		0.596	0.651	0.762	0.619	0.062	0.098	0.098	0.075
Variety	0II	0.383	0.329	0.501	0.060	0.914	0.682	0.682	0.574
Drought stress		0.837	0.533	0.686	0.346	0.358	0.283	0.283	0.071
Variety x Drought stress		0.436	0.850	0.773	0.408	**0.045**	**0.038**	**0.038**	0.675
Variety	SI	**0.003**	**0.008**	**0.001**	0.234	0.373	0.088	0.088	0.623
Drought stress		**0.000**	**0.000**	**0.000**	0.489	**0.003**	0.257	0.257	0.054
Variety x Drought stress		0.460	0.563	0.626	0.689	0.708	0.055	0.055	0.667
Variety	SII	0.103	**0.029**	0.106	0.095	0.307	0.322	0.322	0.555
Drought stress		**0.000**	**0.000**	**0.000**	0.120	**0.000**	**0.000**	**0.000**	**0.000**
Variety x Drought stress		**0.019**	**0.005**	**0.018**	0.078	0.346	0.302	0.302	0.906
Variety	SIII	0.577	0.336	0.783	0.894	0.186	0.253	0.253	0.811
Drought stress		**0.000**	**0.000**	**0.000**	**0.000**	**0.001**	**0.005**	**0.005**	**0.000**
Variety x Drought stress		0.098	0.527	0.617	0.310	**0.044**	**0.092**	0.092	0.807
Variety	RI	0.994	0.471	0.835	0.589	0.640	0.795	0.795	0.359
Drought stress		**0.000**	**0.000**	**0.000**	0.373	**0.000**	**0.000**	**0.000**	0.138
Variety x Drought stress		0.810	0.343	0.692	0.308	0.920	0.786	0.786	0.726
Variety	RII	0.223	0.058	**0.049**	0.058	**0.043**	0.630	0.630	0.192
Drought stress		0.184	**0.004**	0.129	0.291	0.721	0.656	0.656	0.153
Variety x Drought stress		0.387	0.987	0.471	0.067	0.075	**0.049**	**0.049**	0.441

Significant (p<0.05) factors are bold faced.

**Fig 4:**
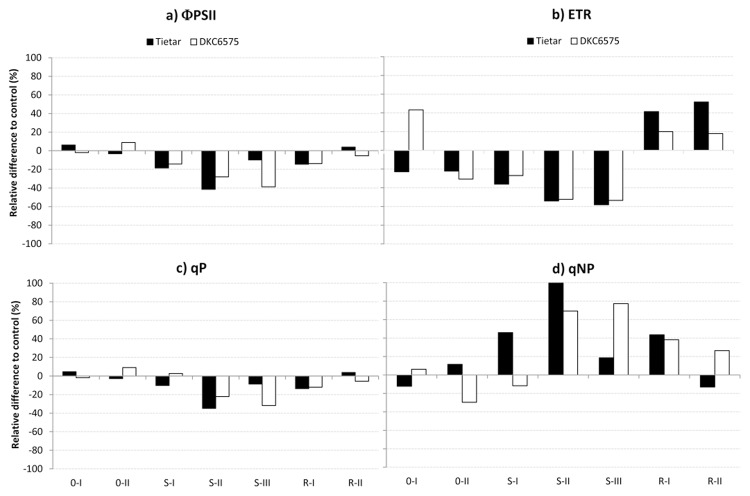
Percentage variation of the drought stress treatment (irrigation 0% of field capacity) with respect to the control (irrigation 100% of field capacity) for selected chlorophyll fluorescence parameters measured in the growth chamber experiment. The straight line at the “0” level represents the control, and histograms represent the relative variations of drought stressed plants.

As for chlorophyll fluorescence, the effective quantum efficiency ΦPSII was slightly, but significantly, reduced in both varieties already after 4 d without irrigation (SI). At SII, water stressed plants displayed a ΦPSII reduction of 41.5% and 28.1% compared with the control, for Tietar and DKC6575, respectively; however, while the ΦPSII reduction partially recovered in Tietar plants at SIII, the ΦPSII for DKC6575 continued to decrease until re-watering (Fig 4, Table 4). Interestingly, a variety x treatment effect was evident at RII for qP and qNP, with DKC6575 showing an incomplete recovery from the stress effect (Fig 4, Table 4). The A/Ci response curves measured at the end of the treatment confirmed that the GM hybrid was more affected by drought than its isogenic non-GM variety. V_c,max_ was only 25% that of the control in DKC6575, and 37% in Tietar. Concurrently, an increase of the CO_2_ compensation point of 1877% in DKC6575 and 316% in Tietar was recorded (data not shown). However, these differences did not result in a between-variety difference in biomass production after drought. Indeed, although water shortage negatively affected leaves, stems and root biomass in both varieties, no significant difference in biomass was evident between the GM and non GM maize variety (Tables 5 and 6).

**Table 5:** Dry biomass (g) of leaves, stems, and roots, of control and drought stressed plants at the end of the drought treatment in the growth chamber experiment.

Variety	Treatment	Leaves (g)	Stems (g)	Roots (g)
Tietar	Non-stressed	23.45 ± 2.84	21.15 ± 3.66	10.93 ± 2.69
	Drought stressed	19.12 ± 2.36	14.38 ± 2.83	7.20 ± 1.36
DKC6575	Non-stressed	23.06 ± 2.52	20.33 ± 4.27	11.15 ± 4.09
	Drought stressed	18.53 ± 2.88	12.85 ± 2.76	6.64 ± 1.40

Values represents mean ± Standard Deviation of n = 7 plants.

**Table 6:** Two-Way ANOVA on dry biomass (g) of leaves, stems, and roots, of non-stressed and drought stressed plants at the end of the drought treatment in the growth chamber experiment.

Factor	Leaves	Stems	Roots
Variety	0.629	0.375	0.867
Drought stress	**0.000**	**0.000**	**0.000**
Variety x Drought stress	0.923	0.787	0.694

Significant (p < 0.05) factors are marked in bold.

### Gene expression analysis by qRT-PCR

Field experiment

**Fig 5:**
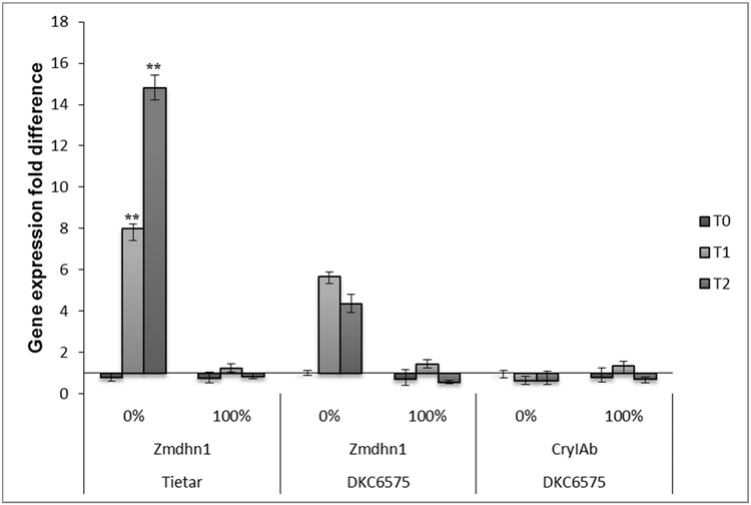
Time course of *Zmdhn1* and *CryIAb* transcription evaluated by qRT-PCR in samples from the Field experiment. Samples of leaves were collected from plants in the Field experiment at the vegetative six-leaves stage (V6) (T0 stage), at the vegetative eight-leaves stage (V8) (T1 stage) and at silking (R1) (T2 stage), all at the same hour of day. 0% plants not irrigated from V6 stage to harvest; 100% plants fully irrigated (control). Relative quantification of gene expression is based on the 2^-ΔΔCt^ method using *Zmabp3* as reference gene. Samples are a pool of four plants that were examined in triplicate. Each value is the mean +/- SD. ** indicate a significant difference at p<0.01 evaluated by ANOVA.

A transcriptional analysis on the following targets: *Zmabp3* (actin binding protein) and *Zm18SrRNA* as reference genes, *Zmdhn1* (dehydrin) as stress responsive gene and *CryIAb*, the transgene, was performed using samples derived as described in Materials & Methods. The results of qRT-PCR analyses are shown in Fig. 5. The expression of Z*mdhn1* increased under drought stress in both varieties at T1 and T2, demonstrating the presence of drought stress in leaves. The *CryIAb* transgene expression was maintained at a high level, showing only small variations under all experimental conditions.

ANOVA showed that *Zmdhn1* transcription level varied significantly in response to stress, but not under control conditions; similarly in Tietar and in DKC6575, while for the transgene *CryIAb* no significant variation was observed between the two conditions.

Growth chamber experiment

The transcriptional analysis regarded the target genes *Zmabp3* (actin binding protein) as housekeeping gene, *Zmdhn1* (dehydrin) as a stress responsive gene and *CryIAb*, the transgene. Expression of each target genes was measured at full irrigation (0I, 0II), during the drought treatment (SI, SII corresponding to 4 and 5 d from the last irrigation), and after the re-irrigation of the S sets (RI, RII corresponding to 2 and 4 d after re-irrigation) in both GM and non-GM varieties.

**Fig 6:**
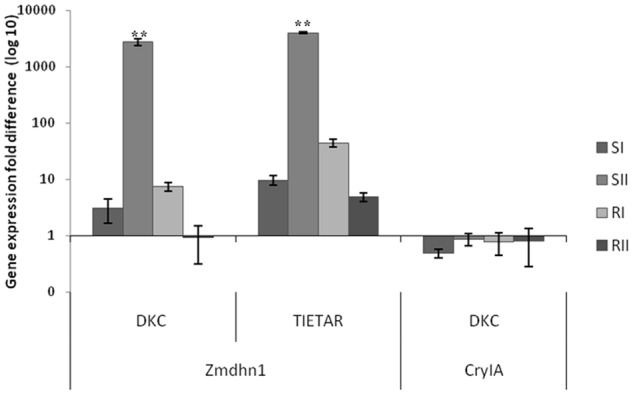
Time course of *Zmdhn1* and *CryIAb* transcription evaluated by qRT-PCR in samples of the Growth chamber experiment. Samples of leaves were collected from plants at full irrigation of all sets (0I, 0II) during the drought treatment (SI, SII, corresponding to 4, and 5 d from the last irrigation of the “S” sets), and after the re-irrigation of the S sets (RI, RII corresponding to 2 and 4 d after re-irrigation). Relative quantification of gene expression is based on the 2^-ΔΔCt^ method using *Zmabp3* as reference gene and samples 0I and 0II as control. Samples are a pool of two plants that were examined in triplicate. Each value is the mean +/- SD. ** indicate a significant difference at p<0.01 evaluated by ANOVA.

As shown in Fig. 6, *Zmdhn1* transcription level was significantly increased in SII corresponding to 5 d of drought stress, while it returned to its control levels during recovery (RI-RII). The mild drought stress at SI did not increase *Zmdhn1* expression significantly. Comparing DKC6575 with Tietar it was evident that *Zmdhn1* expression was significantly higher in Tietar under all conditions tested. In DKC6575, expression of the transgene *CryIAb*, evaluated at the same time intervals, showed no significant differences under all conditions tested.

Analysis of microarray data

The Maize Oligonucleotide microarray was used to measure differences in gene expression levels under drought stress in DKC6575 and in the near-isogenic variety Tietar growing in the field. The gene expression profile of each variety was evaluated at T1 and at T2 for samples fully irrigated and at T2 for samples with drought stress. At T1 the two varieties had a similar expression profile for the set of genes tested with the microarray, as shown in S1 Fig. and S2 Fig. The gene expression profiles for DKC6575 and Tietar were compared both under control conditions and in conditions of drought stress. Genes showing at least a two-fold differential expression were considered for bioinformatic analysis (S1 Table).

**Fig 7:**
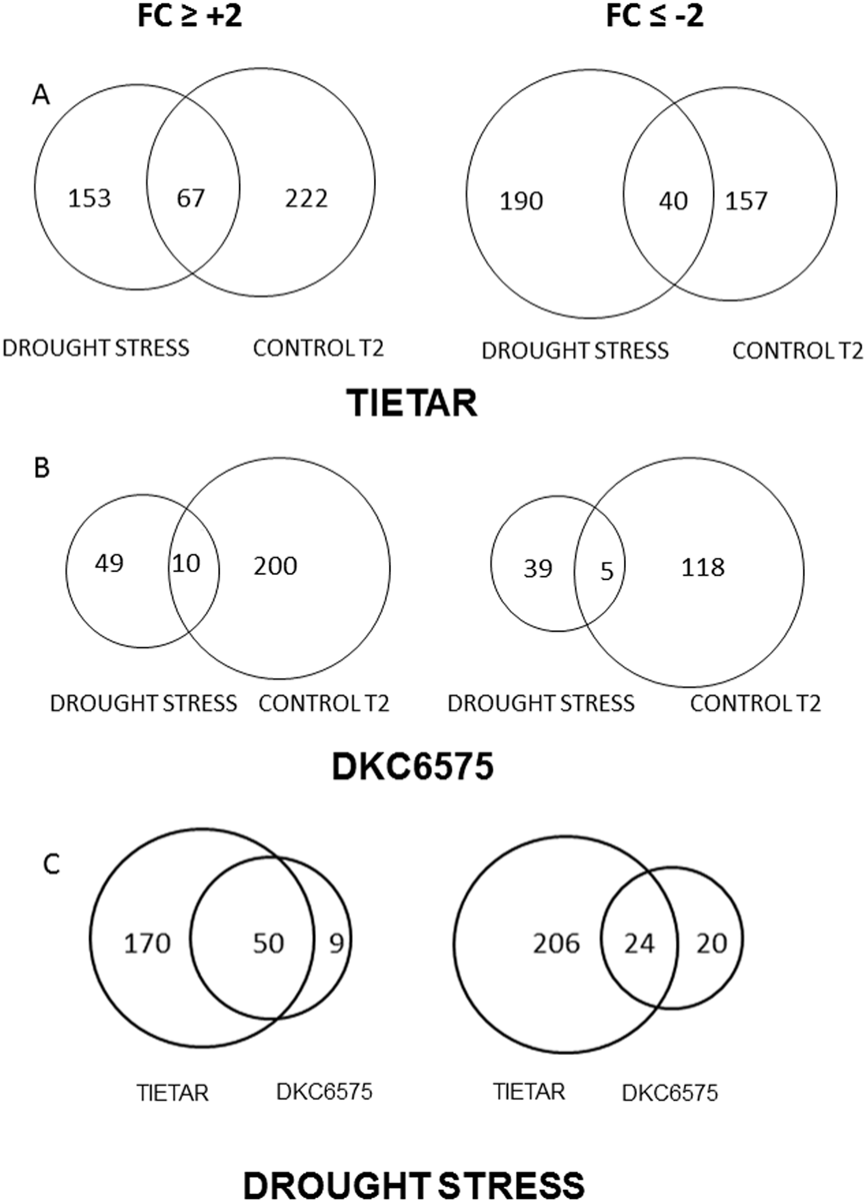
Venn diagrams representing the number of genes differentially expressed based on microarray analysis. Plants grown in the Field experiment were sampled at the developmental stage T2 in the drought stress condition and at T1 and T2 in the control condition. Genes considered are those showing a fold change (FC) ≥±2.

**Fig 8:**
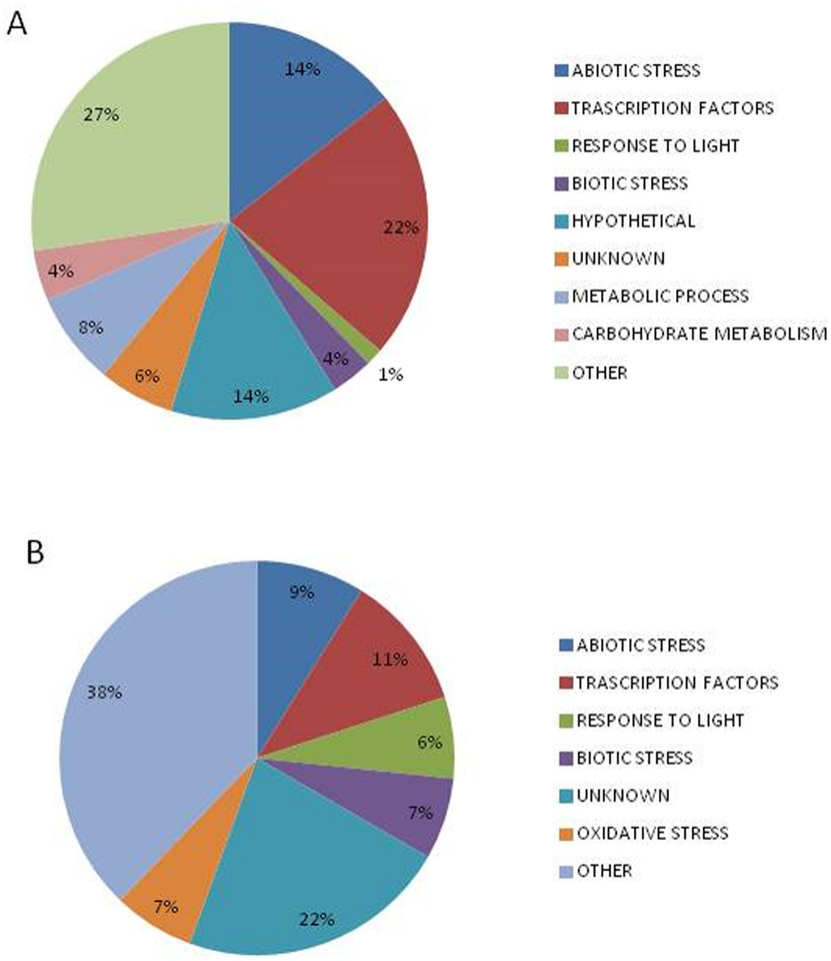
Functional annotation chart of the transcripts up-regulated in Tietar (A) and in DKC6575 (B) in response to drought stress.

**Fig 9:**
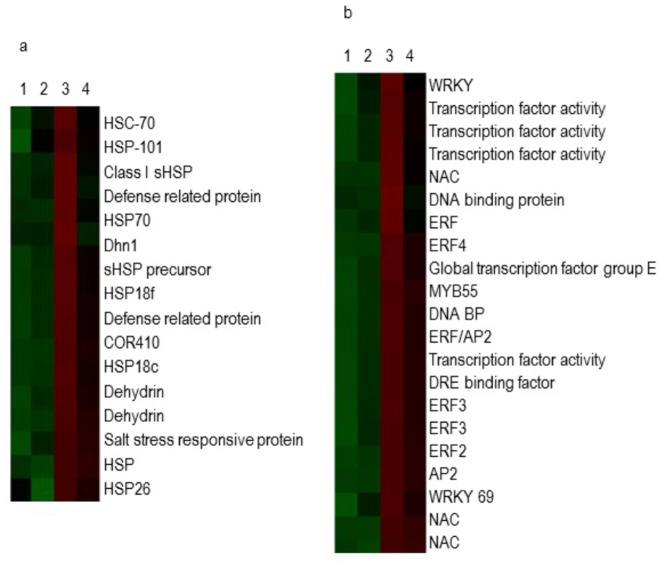
Hierarchical clustering of transcriptional responses of different classes of genes in the two maize genotypes: a) Heat Shock Proteins, Late Embryogenesis Abundant proteins and dehydrins; b) transcription factors. Sample numbers are indicated on the top: 1) DKC6575 at T2 drought stress; 2) DKC6575 at T2 control condition; 3) Tietar at T2 drought stress; 4) Tietar at T2 control condition. A heat map was generated by Java TreeView 1.60 software. Each colored block represents the expression of one gene (labeled on the right) in the indicated sample. Expression signals are converted into color (red, high signal; green, low signal). A ≥2 fold change is shown in red, a fold change ≤0.5 in green and no change in black. Color intensities are proportional to the variation of expression.

In Tietar, a total of 387 genes were down-regulated: 190 genes by drought stress, 157 during development (T2 vs T1), and 40 genes by both stress and development. A total of 442 genes were up-regulated: 153 by drought stress, 222 during development (T2 vs T1), and 67 genes by both (Fig. 7a). In DKC6575, a total of 162 genes were down regulated: 44 transcripts by drought stress, 123 during development (T2 vs T1), and 5 genes by both stress and development. A total of 259 genes were up-regulated: 59 by drought stress, 210 during development (T2 vs T1), and 10 genes by both (Fig. 7b). More genes were differentially regulated during development than in response to water stress, particularly in DKC6575, the GM variety. Differences in number, level of expression and type of responsive genes were observed between the two varieties under water stress. As shown in Fig. 7c, 229 genes were up-regulated by drought stress, 170 in Tietar, 9 in DKC6575, and 50 in both; similarly a total of 250 genes were down-regulated by drought stress; 206 in Tietar, 20 in DC6575 and 24 in both. Changes in gene expression between drought stressed and watered plants were greater for Tietar than for DKC6575. In Fig. 8, charts show the functional annotation of the genes up-regulated in stress conditions for both varieties. Among the genes differentially expressed, genes involved in abiotic stress response represented 14% in Tietar and 11% in DKC6575; genes encoding transcription factors (TFs) 22% and 11% respectively. The expression levels of these genes, as measured with microarray analysis was compared in both varieties under control and drought conditions (Fig. 9). In response to drought, genes encoding Heat Shock Proteins (HSPs), Late Embryogenesis Abundant (LEA) proteins, defence proteins and detoxification enzymes were induced to a greater extent in Tietar than in DKC6575. Similarly, a greater number of genes from different TFs gene families was up-regulated in Tietar, belonging to families previously implicated in stress responses such as members of the AP2/ERF, bZIP, NAC, MYB, and WRKY TF gene families.

## Discussion

A number of papers have been published regarding the possible unintended effects of the introduction and expression of transgenes in different plant species, [23–31]. The majority of these studies were performed under optimal and controlled growth conditions, with plants cultured in vitro, or in growth chambers. However, for a commercial GM variety it is important to verify any unintended effect also under real agricultural conditions, considering possible environmental stress, such as water shortage or high temperature. The purpose here was to compare DKC6575 and Tietar, one GM and one non-GM maize variety, respectively, for physiological and molecular responses to drought, including the expression of the transgene *CryIAb*. Previous studies on Tietar have shown that this variety had a strong growth and yield in response to water availability [32] but, to the best of our knowledge, the drought response of DKC6575 has never been tested before.

### Field experiment

After 20 d of water limitation applied in the field, the reduction of net photosynthesis observed in plants of both maize varieties was driven by stomatal closure, as indicated by the decrease of the Ci/Ca ratio [33]. The increase of WUE under drought, typical of the C4 metabolism [34], as well as maintenance of the maximum quantum yield of primary photochemistry ΦPSII, confirms that mesophyll photosynthetic capacity was unaltered in both varieties [35]. The observed reduction of linear electron transport, ETR, can be considered as a down-regulation mechanism instead, that could be attributed to the concurrent decrease of A, generating an imbalance between NADPH production and its utilization in the Calvin Cycle [36], [37]. Therefore, the varieties Tietar and DKC6575 showed a similar strategy to cope with mild drought conditions: i.e. minimizing of water loss through stomatal closure [37]. Nevertheless, the presence of multiple stress factors can influence plant responses to drought under field conditions, and that such responses are generally more complex than those measured under controlled environment conditions [38].

Studies on crop plants with differences in abiotic stress tolerance have shown a positive correlation between dehydrins gene expression and plant stress tolerance [39], [40], and for this reason it is considered that their expression can be used as a marker of stress. The expression of *Zmdhn1*, encoding a dehydrin, was analysed to verify whether the irrigation regime was really determining a drought stress and the results demonstrated a significant increase in the transcription of this gene, as expected.

The expression of *CryIAb* tested whether the irrigation regime utilized but no significant difference in transcription of the transgene was observed even though a decrease, but not significant, was observed in the stressed samples. Some evidence [41], [42] showed that severe environmental cues, including water stress, can affect the overall development and performance of maize. Comparing Bt and non-Bt varieties in terms of temperature and drought stress resistance, a 9% yield advantage of Bt over non-Bt plants was reported when the European corn borer (ECB) pressure was high on plants. In the absence or under low ECB infestation, Bt varieties performed similarly to non-Bt conventional counterparts. It was also demonstrated that incorporation of the Bt gene into corn hybrids provided a higher level of protection against ECB, but had little or none agronomic advantage. In transgenic Bt cotton, water stress did not significantly decrease Bt efficacy in any of the plant organs tested [43]; in transgenic peas containing a seed specific α-amylase inhibitor [44], its level was not influenced by water stress, but was reduced in a high-temperature condition affecting also the protective capacity of the transgenic peas against pea weevil.

There is evidence [45] that mycotoxin levels may be affected indirectly by a decrease of plant health as resulting from insect damage. Indeed results from field trials are not consistent, confirming that fumonisin contamination can be reduced by Bt expression, whereas the data on aflatoxin are, at present, inconclusive. It is suspected that a potential strategy to control mycotoxin contamination, could be to combine Bt technology with biological control using non-toxigenic *A*. *flavus* isolates [46].

In this paper, a genome wide analysis of the transcriptional profile through microarray demonstrated in Tietar the induction of several classes of genes involved in abiotic and biotic stress response. The induction of genes encoding for HSPs (HSP18, HSP26, HSP70, HSP101) could be related to the role of these proteins within the cytoplasm to prevent protein aggregation and assist protein refolding, helping to protect from the detrimental effects of stress [47]. Induction of these genes in response to drought was reported in previous studies in maize [48], and in other cereals [39], [49], [50].

The induction of genes encoding LEA proteins (dhn1, dhn2, COR14) was an expected event in condition of drought stress, as these proteins are induced during physiological desiccation in maturing embryo, but also when plants are exposed to stresses resulting in a cellular dehydration (e.g., drought, osmotic stress, salinity, temperature), and they accumulate to higher amounts in all vegetative tissues [51]. The role of dehydrins, however, could be part of defence mechanisms against pathogen infection, usually present during periods of water scarcity. Evidence exists of an induction by wounding, which is a common stress due to insects attack, but which is also considered a dehydration stress, as the cellular damage can lead to water loss [40].

Increase in the level of transcripts in response to drought requires the participation of components of signalling pathways that activate transcription and /or RNA stabilisation. In particular it requires the participation of several TFs some of which are transcriptionally activated during the stress. Most of these TFs belong to large gene families such as AP2/ERF, bZIP, NAC, MYB, and WRKY [52]. As evidenced in Fig. 8, the number of TFs whose expression was increased during drought stress, was proportionally much higher in Tietar than in DKC6575. In Tietar, several TFs families were represented among those genes whose response is modified by drought, whereas in DKC6575 only few members of TFs were up-regulated.

In Tietar, the up-regulation regarded a greater number of genes encoding HSPs, LEA, and defence proteins, and of genes encoding TFs known to be involved in improving abiotic and biotic stress tolerance. Similar observations have been reported in a study with maize landraces differing in drought stress tolerance when compared at the transcriptional level [48]. The more tolerant genotypes have a greater capacity to rapidly modulate more genes under drought stress than the more susceptible landraces. In particular, modulation of a greater number of differentially expressed genes and of different TF gene families has been considered an important characteristic of the more tolerant genotypes.

By the evidences provided by our study, in which DKC6575 and Tietar were grown in the field under water stress regimes, DKC6575 demonstrated to be less responsive than Tietar in terms of gene regulation. Whether this functional genomic difference was correlated to some phenotypic difference in drought stress tolerance is discussed below.

### Growth chamber experiment

In the growth chamber experiments, the physiological effect of drought stress on potted plants of both varieties was more severe than that observed under field conditions. Interestingly, after 5 days from the last irrigation (SII), Tietar plants showed a higher gas exchange reduction than DKC6575. This early stomatal closure is considered as an indication of a better degree of drought stress tolerance, since it decreases hydraulic gradients allowing plants to save soil water for a longer time [38]. The reduction of gas exchanges was also accompanied by a significant reduction of maximum quantum yield of primary photochemistry ΦPSII, of ETR and of photochemical quenching qP, while the qNP was increased, confirming that also non-stomatal limitations to photosynthesis were occurring [37], [53]. In particular, the decrease of ΦPSII may reflect a relative increase in alternative processes for electron consumption under conditions of limited CO_2_ assimilation, as found in other C4 and C3 species [35], [54]. The decrease of ΦPSII at SIII in DKC6575 plants, as well as the alterations of qP and qNP persisting until 2 days after re-irrigation for this variety, seem to support a lower tolerance to severe drought conditions in the GM variety. Considering the expression of a typical drought stress induced gene such as *Zmdhn1*, the level of its transcription was related with the stress perception. In Tietar the induction of *Zmdhn1* occurred in the early phase of stress, differently from DKC6575. This observation reinforced the role of dehydrins as markers of the stress. Biomass production was comparable between DKC6575 and Tietar supporting previous evidences in which comparing commercial GM maize varieties and their near-isogenic non-GM lines no yield advantage of GM plants was observed in the absence of the pest infestation [42].

## Conclusion

In conclusion, this study has highlighted that: i) although the main photosynthetic parameters were affected by drought to a similar extent in both the GM and non-GM varieties, under controlled environmental conditions DKC6575 was demonstrated to have a greater sensitivity to stress in the early phase with respect to Tietar; ii) a whole genome transcriptomic analysis demonstrated that the water deficit regimes determined the up- and down-regulation of many genes, but with an up-regulation of stress-responsive genes to a greater extent in Tietar, suggesting more efficient drought responses in this genotype than in DKC6575; iii) the expression of the transgene *CryIAb* was not influenced by the water regime, being expressed at a constant level, suggesting that any eventual greater sensitivity to drought stress in the GM variety did not concern the level of transgene expression, which was stable through conditions. These results cannot be ascribed to a substantial genetic background difference between DKC6575 and Tietar (which are commonly indicated as near isogenic in other papers) because the global gene expression pattern in control condition (T1) was very similar (S1 and S2 Figs.) between the two.

This study calls for a better assessment of the ecological behaviour of GM plants under different levels of environmental stress, in comparison to their near-isogenic non-GM varieties. However, the mechanism underlying the less efficient drought response in DKC6575 remains unknown, and it is not possible to attribute the observed differences to the transgene insertion. Previous studies have shown that several pairs of maize varieties obtained from classical breeding displayed different levels of metabolomic divergence, that were consistently higher than those found between GM/non-GM variety pairs [30] [55]-[57]. The transgenic event may have unintended effects on plant gene networking, potentially affecting plant-environment interaction in the field, but before any generalization can be drawn, further studies are needed to test more varieties and more stress factors other than those considered in this study.

## Supporting Information

S1 Fig Hierarchical clustering of global gene expression. Global gene expression was obtained through microarray analysis of the Tietar and DKC6575 samples taken from plants grown in the Field experiment at the developmental stage T2 in the drought stress condition and at T1 and T2 in the control condition.
(DOCX)

S2 Fig Principal Component Analysis from the gene expression data set generated from the Tietar and DKC6575 samples. Each circle represents a sample at different stage. T1, control condition for Tietar and DKC6575 (CONT1T, CONT1D) are represented as red circles; T2, control condition for Tietar and DKC6575 (CONT2T, CONT2D) are represented as blue circles, T2, drought stress condition for Tietar and DKC6575 (STRESST, STRESSD) are represented as yellow circles.
(DOCX)

S1 Table List of genes showing differential expression in Tietar and DKC6575. (XLSX)
