# Supplementary material for: Comparison of Drought Stress Response and Gene Expression between a GM Maize Variety and a Near-Isogenic Non-GM Variety
Source: PLoS One. 2015 Feb 18;10(2):e0117073. doi: 10.1371/journal.pone.0117073 (PMC4333122; doi:10.1371/journal.pone.0117073)
Supplement: S1 Fig — Global gene expression was obtained through microarray analysis of the Tietar and DKC6575 samples taken from plants grown in the Field experiment at the developmental stage T2 in the drought stress condition and at T1 and T2 in the control condition. (DOCX) [file pone.0117073.s001.docx]

**Figure S1. Hierarchical clustering of global gene expression.** Global gene expression was obtained through microarray analysis of the Tietar and DKC6575 samples taken from plants grown in the Field experiment at the developmental stage T2 in the drought stress condition and at T1 and T2 in the control condition.

**
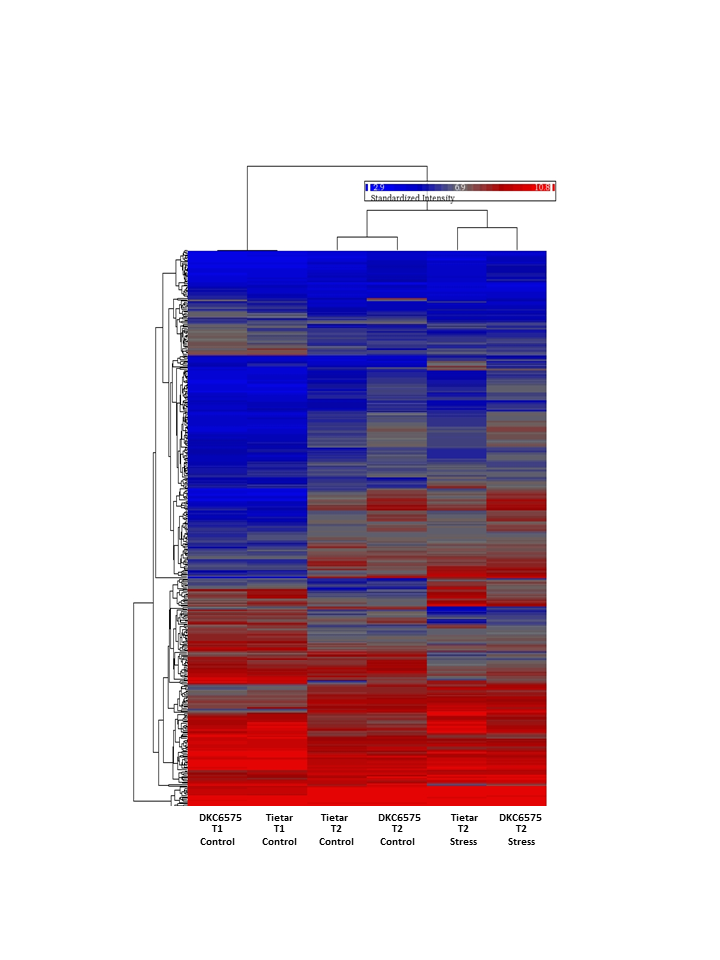
**
