# Supplementary material for: Comparison of Drought Stress Response and Gene Expression between a GM Maize Variety and a Near-Isogenic Non-GM Variety
Source: PLoS One. 2015 Feb 18;10(2):e0117073. doi: 10.1371/journal.pone.0117073 (PMC4333122; doi:10.1371/journal.pone.0117073)
Supplement: S2 Fig — Each circle represents a sample at different stage. T1, control condition for Tietar and DKC6575 (CONT1T, CONT1D) are represented as red circles; T2, control condition for Tietar and DKC6575 (CONT2T, CONT2D) are represented as blue circles, T2, drought stress condition for Tietar and DKC6575 (STRESST, STRESSD) are represented as yellow circles. (DOCX) [file pone.0117073.s002.docx]

**Figure S2.** **Principal Component Analysis from the gene expression data set generated from the Tietar and DKC6575 samples.** Each circle represents a sample at different stage. T1, control condition for Tietar and DKC6575 (CONT1T, CONT1D) are represented as red circles; T2, control condition for Tietar and DKC6575 (CONT2T, CONT2D) are represented as blue circles, T2, drought stress condition for Tietar and DKC6575 (STRESST, STRESSD) are represented as yellow circles.


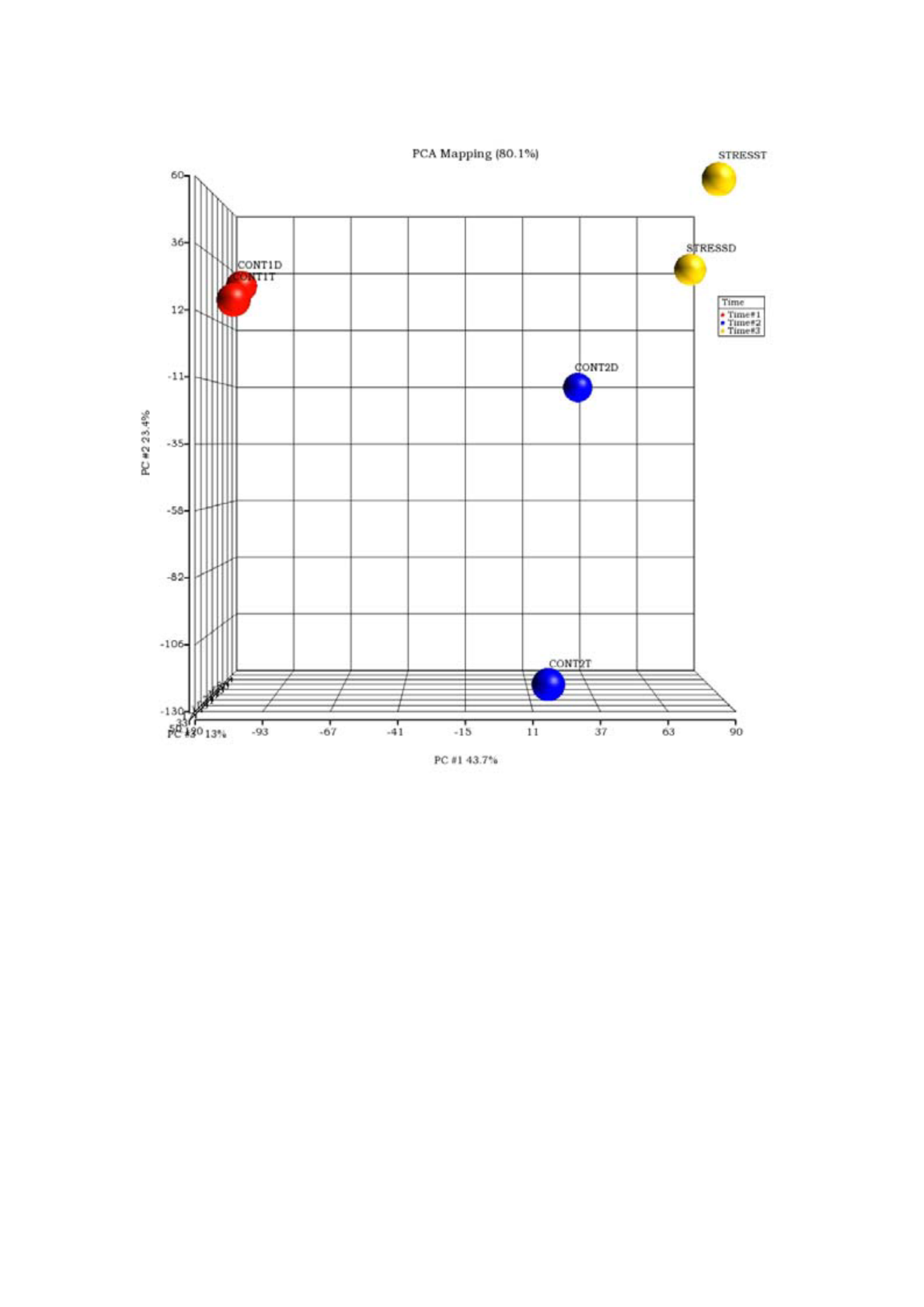


D
